# The 3'-5' exoribonuclease Dis3 regulates the expression of specific microRNAs in *Drosophila* wing imaginal discs

**DOI:** 10.1080/15476286.2015.1040978

**Published:** 2015-04-18

**Authors:** Benjamin P Towler, Christopher I Jones, Sandra C Viegas, Patricia Apura, Joseph A Waldron, Sarah K Smalley, Cecilia M Arraiano, Sarah F Newbury

**Affiliations:** 1 Brighton and Sussex Medical School; Medical Research Building; University of Sussex; Falmer, Brighton, UK; 2 Instituto de Tecnologia Química e Biológica; Universidade Nova de Lisboa; Oeiras, Portugal

**Keywords:** Dis3/Taz, *Drosophila* development, exoribonuclease, imaginal discs, miRNAs, RNA stability, RNA degradation

## Abstract

Dis3 is a highly conserved exoribonuclease which degrades RNAs in the 3'-5' direction. Mutations in Dis3 are associated with a number of human cancers including multiple myeloma and acute myeloid leukemia. In this work, we have assessed the effect of a Dis3 knockdown on *Drosophila* imaginal disc development and on expression of mature microRNAs. We find that Dis3 knockdown severely disrupts the development of wing imaginal discs in that the flies have a “no wing” phenotype. Use of RNA-seq to quantify the effect of Dis3 knockdown on microRNA expression shows that Dis3 normally regulates a small subset of microRNAs, with only 11 (10.1%) increasing in level ≥2-fold and 6 (5.5%) decreasing in level ≥2-fold. Of these microRNAs, *miR-252–5p* is increased 2.1-fold in Dis3-depleted cells compared to controls while the level of the *miR-252* precursor is unchanged, suggesting that Dis3 can act in the cytoplasm to specifically degrade this mature miRNA. Furthermore, our experiments suggest that Dis3 normally interacts with the exosomal subunit Rrp40 in the cytoplasm to target *miR-252–5p* for degradation during normal wing development. Another microRNA, *miR-982–5p*, is expressed at lower levels in Dis3 knockdown cells, while the *miR-982* precursor remains unchanged, indicating that Dis3 is involved in its processing. Our study therefore reveals an unexpected specificity for this ribonuclease toward microRNA regulation, which is likely to be conserved in other eukaryotes and may be relevant to understanding its role in human disease.

## Introduction

Ribonucleases are enzymes that degrade or process RNAs. Since the level of a particular RNA within a cell is the result of a balance between RNA synthesis and degradation, ribonucleases are key components in the regulation of gene expression. There is increasing evidence that mutations in genes encoding ribonucleases can result in defects in cellular processes, organism viability and cancer, suggesting that they play a key role in development and differentiation.^1-4^ Furthermore, recent data has shown that ribonucleases can target specific RNAs, which can in turn affect specific cellular pathways.^5,6^ However, the effects of ribonucleases on biological functions and the RNAs targeted by specific ribonucleases in whole organisms or tissues are not well understood.

This paper focuses on Dis3/Tazman, a highly processive ribonuclease that belongs to the RNaseII/RNB family of hydrolytic 3′-5′ exonucleases.^7^ In the yeast *S. cerevisiae*, only one form of Rrp44/Dis3 is present, whereas there are 2 paralogues present in *Drosophila melanogaster* (*Dis3* and *Dis3L2*) and 3 in humans (*DIS3, DIS3L1* and *DIS3L2*).^3,8^ Dis3 and Dis3L1 are known to associate with the exosome, a 9-subunit core complex which serves to channel the RNA through its central core to the catalytic subunit.^9-12^ In eukaryotes, Dis3 proteins are similar in domain structure and include a CR3 motif, 2 cold-shock (OB) domains, the RNB domain, an S1 domain and 2 putative nuclear localization sequences. The cold-shock domains and S1 domains are ancient folds that non-specifically bind RNA, while the exonucleolytic activity is provided by the RNB domain. Dis3 and Dis3L1 also include a PIN (PilT N-terminus) domain located at the N-terminus between the CR3 motif and the cold-shock domains. The PIN domain in Dis3 is known to catalyze endonucleolytic RNA decay as well as tethering Dis3 to the exosome.^13^ Although Dis3L1 includes a PIN domain which results in its association with the core exosome, this PIN domain does not have endonucleolytic activity.^11,14,15^ Dis3L2 has a similar domain structure to Dis3 and Dis3L1 but does not include a PIN domain, does not associate with the exosome and is responsible for an alternative degradation pathway involving uridylation of RNA substrates.^8,16-18^ Previous reports using human and *Drosophila* tissue culture cells or *Drosophila* oocytes have shown that Dis3 is predominantly located in the nucleus but is also present in the cytoplasm.^7,11,19,20^ In human tissue culture cells DIS3L1 and DIS3L2 are restricted to the cytoplasm.^8,11,12,17^

Dis3 has essential functions in the cell as null mutations result in lethality.^21-24^ Loss of function by hypomorphic mutations or by RNA knockdown using siRNAs also results in reduced cell survival.^24^ Global screens have revealed that mutations or aberrant expression of Dis3 are often associated with human cancers such as multiple myeloma, medulloblastoma, acute myeloid leukemia and melanoma.^25-28^ The effects of Dis3 on cell survival and proliferation are most likely due to increased amounts of target RNAs which, directly or indirectly, perturb cellular functions. The reported targets of Dis3 reflect its predominantly nuclear location and include human PROMPTS (promoter upstream transcripts) or yeast CUTs (cryptic unstable transcripts), excised introns, pre-mRNAs and pre-tRNAs as well as snRNAs and snoRNAs.^22,24,29^ Dis3 also plays a role in microRNA (miRNA) biogenesis, in that it is also involved in the maturation of miRNAs derived from introns (mirtrons) such as *Drosophila miR-1017* and the trimming of pre-miRNA 3′ ends.^30^ In *Drosophila* S2 cells, depletion of Dis3 using RNA interference results in increases in RNAs encoding cell cycle related proteins, whereas in whole larvae, Dis3 knockdown results in the increase of a discrete set of transcripts at different stages of development.^31,32^ However, the effect of Dis3 on the development of a specific tissue or on mature miRNAs in whole organisms has not yet been addressed.

In this paper, we have, for the first time, used genome-wide profiling (miRNA-seq) on *Drosophila* wing imaginal discs to assess the effect of Dis3 on mature miRNA gene expression. We show that Dis3 knockdown within wing imaginal discs has a profound effect on wing differentiation and on developmental timing. We also show that, surprisingly, Dis3 knockdown affects the levels of a small set of miRNAs, whereas the levels of most miRNAs are unaffected. For one miRNA, the levels of mature miRNA increase while the levels of its precursor remain unchanged, suggesting that Dis3 is involved in the degradation of this miRNA in the cytoplasm.

## Results

### Knockdown of Dis3 in the wing imaginal discs results in severe wing defects and delayed development

**Figure 1.**
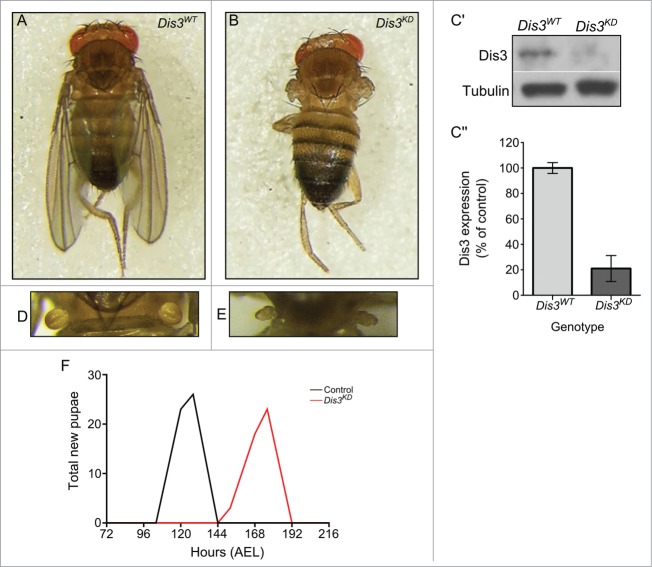
Knockdown of Dis3 in the wing imaginal disc results in severe developmental phenotypes. Knockdown of Dis3 in the wing pouch of the wing imaginal disc results in a “no wing” phenotype at 100% penetrance (*Dis3*^*KD*^) (**B**) when compared to a *UAS-Dis3*^*RNAi*^ parental control male fly (*Dis3*^*WT*^) (**A**). (**C**) Knockdown of Dis3 protein in *nub-GAL4/+; UAS-Dis3*^*RNAi*^*/+* wing imaginal discs by Western blotting. *Dis3*^*WT*^ genotype: ; *nub-Gal4;*. (**C**) Dis3 expression is knocked down to 20% of parental controls in wing imaginal discs when *UAS-Dis3*^*RNAi*^ is driven by *nub-GAL4*. '*Dis3*^*WT*^' includes parental genotypes ; *nub-Gal4 ;* and *;; UAS-Dis3*^*RNAi*^. n ≥ 3, p = 0 .0009, error bars show standard error. (**D and E**) *nub-GAL4/+; UAS-Dis3*^*RNAi*^*/+* flies also show a severe lack of haltere development (**E**) (100% penetrance) compared to parental control flies (**D**). (**F**) Dis3 knockdown progeny (*nub-GAL4/+ ; UAS-Dis3*^*RNAi*^*/+)* are delayed in larval development by 40 hours (red) when compared to parental controls (black – *UAS-Dis3*^*RNAi*^ and *nub-GAL4*) (n ≥ 23 ).

**Figure 2.**
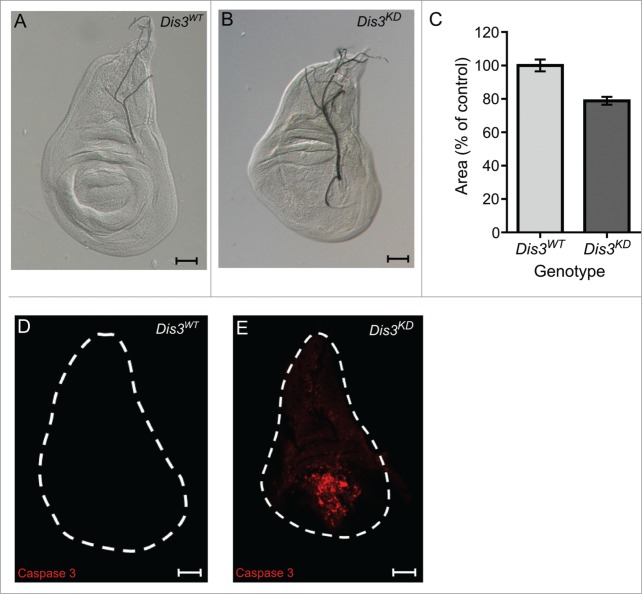
Dis3 knockdown in the wing pouch of the wing imaginal discs results in small wing discs and extensive apoptosis. (**A and B**) Dis3 knockdown (*nub-GAL4/+ ; UAS-Dis3*^*RNAi*^*/+)* late L3 wing imaginal discs (**B**) are smaller than parental control (*Dis3*^*WT*^ is ;; *UAS-Dis3*^*RNAi*^) wing imaginal discs (**A**) (scale bar=50μm). (**C**) Quantitation of *nub-GAL4* driven Dis3 knockdown wing imaginal discs shows that they are 23% smaller than parental control discs ('*Dis3*^*WT*^' includes parental genotypes ; *nub-GAL4* ; and ;;*UAS-Dis3*^*RNAi*^*)*. (n ≥ 21, *P* < 0.0001, error bars represent standard error). (**D and E**) Knockdown of Dis3 in the wing pouch results in apoptosis specifically localized within the wing pouch region (**E**) while parental control discs show very little apoptotic activity (**D**). (n ≥ 26, scale bar = 50 μm).

In order to determine the effect of Dis3/Taz on miRNA expression in imaginal discs, we first analyzed the effect of Dis3 depletion on development of this tissue. As expected, knockdown of Dis3 using ubiquitous GAL4 drivers and *UAS-Dis3*^*RNAi*^ results in complete lethality at or prior to the L2 larval stage (**Table 1**). Knockdown of Dis3 over the entire wing imaginal disc using the *69B-GAL4* driver also results in lethality at the L2 or L3 stage (**Table 1**). However, restricting knockdown of Dis3 to the wing pouch area of the disc, using the *nubbin-GAL4* driver, results in a severe “no wing” phenotype where there is a complete absence of wing blade development (100% penetrance) (**Fig. 1A and B**). In addition to the absence of wing development, late L3 imaginal discs were 77% the size of parental control discs (**Fig. 2A-C**). Staining with Anti-activated Caspase 3 indicates extensive apoptosis specifically in the region of Dis3 depletion (**Fig. 2D-E**). This result indicates that Dis3 is required for viability of cells within the wing pouch and also provides an explanation for the reduced area of the wing imaginal discs. Abnormal haltere development was also observed (**Fig. 1D-E**) as *nubbin-GAL4* also drives expression in these discs.^33^ Although knockdown of Dis3 in the wing pouch (**Fig. 1C**) does not result in lethality, the *nubbin-GAL4*; *UAS-Dis3*^*RNAi*^ flies were delayed in larval development by 40 hours at 25^°^C (**Fig. 1F**). In agreement with these results, knockdown of Dis3 using the *pannier-GAL4* driver to drive expression in tip of the wing disc, which goes on to form the dorsal part of the thorax, results in a severe cleft thorax phenotype (penetrance 100%) (**Fig. S1**). These experiments show that Dis3 is essential for the development of wing imaginal discs into wing and thoracic tissues.

**Table 1.** Stages of lethality observed following ubiquitous knockdown of Dis3

GAL4-Driver	Region of Expression	Stage of Lethality (GD11917 v35090)	Stage of Lethality (KK101473 VIE-260B)
*Tubulin-GAL4*	Ubiquitous	L2	L2
*Daughterless-GAL4*	Ubiquitous	L2	L2
*69B-GAL4*	Ectoderm and wing, haltere, ventral thoracic and eye discs	L2	L3 after 2 week delay

### Global miRNA profiling experiments using RNA-seq reveal that a small subset of specific miRNAs change in expression upon Dis3 depletion

To determine the effect of Dis3 on miRNA expression in the wing imaginal disc we used the *nubbin-GAL4* driver together with *UAS-Dis3*^*RNAi*^ as the limited knockdown of Dis3 in the wing pouch provides a strong phenotype and also suitable tissue for collection for global miRNA profiling. Controls comprised the 2 parental stocks used to generate the Dis3 knockdown flies (*;nub-GAL4;* and ;;*UAS-Dis3*^*RNAi*^). Knockdown of *dis3* RNA, using this shRNA system, resulted in 80% knockdown at the protein level within wing imaginal discs (**Fig. 1C**), even though knockdown only occurs in the wing pouch region of the disc. Total RNA was prepared from 2 biological replicates of 60 wing imaginal discs per genotype and sent to ARK genomics, Roslin Institute, Edinburgh for small library preparation and RNA-sequencing. The sequencing output was mapped to miRBase release 19 to identify the known miRNAs present within each sample.

**Figure 3.**
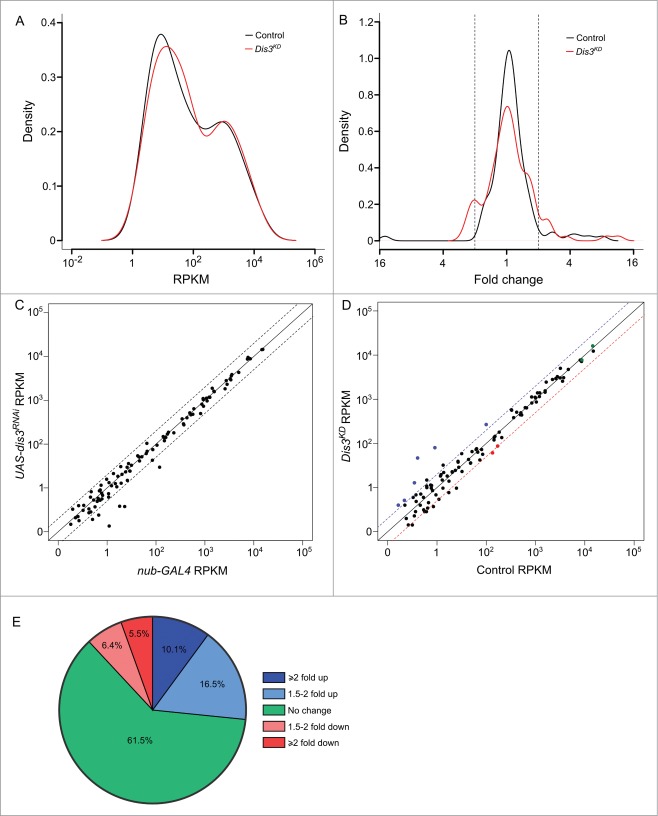
(See previous page). Expression patterns of miRNA in wing imaginal discs. (**A**) Kernel density plot of the general distribution of the 109 detected miRNAs indicates 2 peaks of miRNA expression. Knockdown of Dis3 results in a shift in the poorly expressed miRNAs (left hand peak) to a higher level of expression compared to the more highly expressed miRNAs which do not change (right hand peak). (**B**) Distribution of fold changes between both parents (Control) and grouped parents vs Dis3 knockdown (*Dis3*^*KD*^). Knockdown of Dis3 results in the emergence of peaks around +/− 2-fold change. Dotted vertical lines represent +/− 2-fold change. (**C**) Comparison of miRNA RPKMs between the 2 parental controls shows high similarity. Dotted lines show +/− 2-fold change. (**D**) Comparison of miRNA RPKM between grouped parental controls (Control) and Dis3 knockdown (*Dis3*^*KD*^) wing imaginal discs. Blue and red dotted lines represent +2-fold change and −2-fold change respectively. Selected miRNAs that increase or decrease in expression following Dis3 knockdown are colored in blue and red respectively. Selected miRNAs that remain unchanged are highlighted in green. (**E**) 61.5% of miRNAs in the wing imaginal disc do not change in expression following the knockdown of Dis3. 26.6% of miRNAs increase in expression >1.5-fold (10.1% ≥ 2-fold). 11.9% of miRNAs decrease in expression >1.5-fold (5.5% ≥ 2-fold).

Of the 426 previously annotated miRNAs in *Drosophila* (miRBase 19), 109 were detected that had >1 read in all samples. Duplicates of each sample clustered together (indicating similar miRNA expression profiles) while the 2 parental samples were more similar to each other than they are to the knockdown samples (**Fig. S2**). Expression of 101 (92.7%) miRNAs, normalized to the total number of reads, were similar between parental stocks; those miRNAs that differed more than 2-fold between the parental genotypes were removed from the analysis. To investigate how the levels of each miRNA changed in expression upon Dis3 depletion, the number of reads per miRNA were normalized to the total number of aligned reads within the sample (RPKM). The normalized read count was then compared between control and knockdown samples. Interestingly, the overall expression profiles of miRNAs in wing discs show a bimodal distribution with the highly expressed group (**Fig. 3A**; right hand peak) showing 100 times greater expression than the lowly expressed group (left hand peak). Overall, 67 (61.5%) miRNAs did not change in expression between knockdown and control samples showing that Dis3 depletion does not result in global increases or decreases in miRNA levels (**Fig. 3B and E**). Depletion of Dis3 results in changes in expression of a subset of miRNAs, with 29 (26.6%) increasing ≥1.5-fold and 13 (11.9%) decreasing ≥1.5-fold. These data also show that knockdown of Dis3 affects the expression of poorly expressed miRNAs more than that of highly expressed miRNAs (**Fig 3A and D**), suggesting that Dis3 is normally involved in maintaining these miRNAs at low levels. Dis3 could act on these miRNAs either directly (e.g. by degradation of the miRNA by Dis3) or indirectly (e.g., by control of an mRNA target that subsequently affects the levels of one or more miRNAs).

We have previously published information on the abundance of miRNAs in wing imaginal discs obtained from microarray analysis.^5^ We are now presenting, for the first time, data showing the abundance of miRNAs in wing imaginal discs using the more sensitive and unbiased RNA-seq analysis (**Fig. S3**). A total of 109 mature miRNAs were detected (with >1 read per sample), with the most abundant miRNA, *miR-9a-5p*, roughly 12,000-fold more abundant than the levels of the least abundant miRNA reliably detected (*miR-307a-3p*). These results are similar to that of our previous study using microarrays^5^ in that the highly expressed miRNAs according to microarray analysis are also found to be most abundant in the more sensitive RNA-seq analysis. This analysis also confirms that the miRNAs that change ≥2-fold in levels upon Dis3 depletion are also those that are normally expressed at low levels in wing imaginal discs, suggesting that Dis3 plays a role in keeping the levels of these miRNAs low under normal conditions (**Fig. S3**).

**Figure 4.**
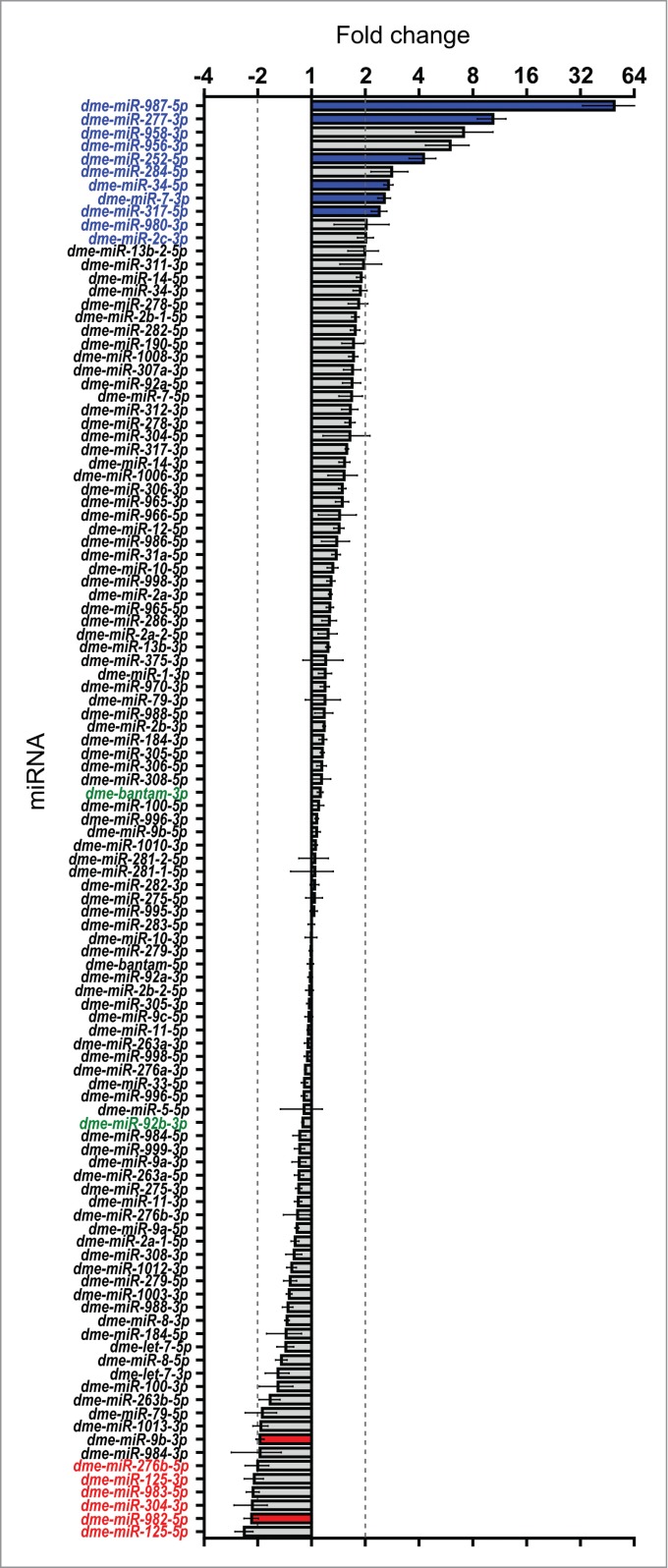
Effect of Dis3 knockdown on all wing imaginal disc miRNAs detected above threshold levels. Fold changes (*Dis3*^*KD*^ vs grouped parental controls) from miRNA-seq data of the 109 detected miRNAs in the wing imaginal disc. *miR-987–5p* shows the greatest increase in expression (49.5-fold) while *miR-125–5p* shows the greatest decrease in expression (−2.4-fold). miRNAs that increase ≥2-fold are highlighted in blue font and those selected for further analysis are shown with blue bars. miRNAs that decrease ≥2-fold are highlighted in red font and those selected for further analysis shown with red bars. Selected miRNAs that remain unchanged are highlighted in green font. Dotted lines represent +/−2-fold changes. (n ≥2, error bars represent standard error).

The effect of Dis3 depletion on miRNA levels in wing imaginal discs is shown in **Figure 4**. These data demonstrate that 11 miRNAs are increased ≥2-fold in levels (blue text). Of these, 5 miRNAs (*miR-987–5p, miR-277–3p, miR-958–3p, miR-956–3p, miR-252–5p*) were upregulated ≥4-fold in Dis3-depleted discs. In contrast, 6 miRNAs were decreased ≥2-fold in levels (red text) with no miRNA downregulated ≥4-fold. The majority of miRNAs (92) show changes of ≤2-fold upon Dis3 depletion. These data show that Dis3 normally represses the expression of a small subset of miRNAs and induces (directly or indirectly) the expression of even fewer miRNAs.

### RNA-seq data reveals a novel miRNA, *miR-11182-3p*, which is conserved in the red flour beetle (*Tribolium castaneum*)

**Figure 5.**
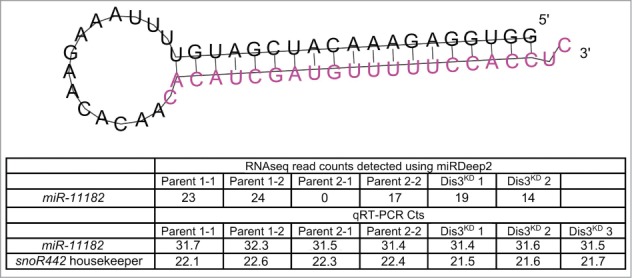
Identification of a novel mature miRNA in the wing imaginal disc. Using miRDeep2 we identified a novel miRNA of moderate expression in the wing imaginal discs. The predicted pre-miRNA forms a hairpin loop secondary structure with the novel miRNA being situated at the 3' end (shown in pink). The novel miRNA was detected at moderate levels using miRNA-seq in all but one sample. Validation of the miRNA using qRT-PCR shows that this novel miRNA is expressed at similar levels to *miR-277–3p* in all samples.

The power of RNA-seq is illustrated by our discovery of a new miRNA, *miR-11182*, which is expressed in wing imaginal discs (**Fig. 5**). This miRNA was identified by use of the program miRDeep2, which scans the RNA-seq data for sequences that map to predicted miRNA precursors that are able to be generated by Dicer cleavage during miRNA biogenesis.^34^ The predicted *miR-11182* precursor is located at 2R:14067655–2R:14067710, within the 5'UTR of *CG5726*. *miR-11182-3p* is similar to *miR-3882–5p* in the red flour beetle (*Tribolium castaneum;* 3 mismatches, score 63, E-value 5.1) suggesting that it is a biologically relevant miRNA. We also validated the presence of *miR-11182-3p* using a custom TaqMan qRT-PCR assay. The assay showed strong detection of the miRNA sequence while no signal was observed in no RT controls. However, according to both the RNA-seq and TaqMan qRT-PCR experiments, this novel miRNA does not change significantly in levels in Dis3-depleted cells compared to parental controls.

### Verification of RNA-seq results using quantitative RT-PCR

**Figure 6.**
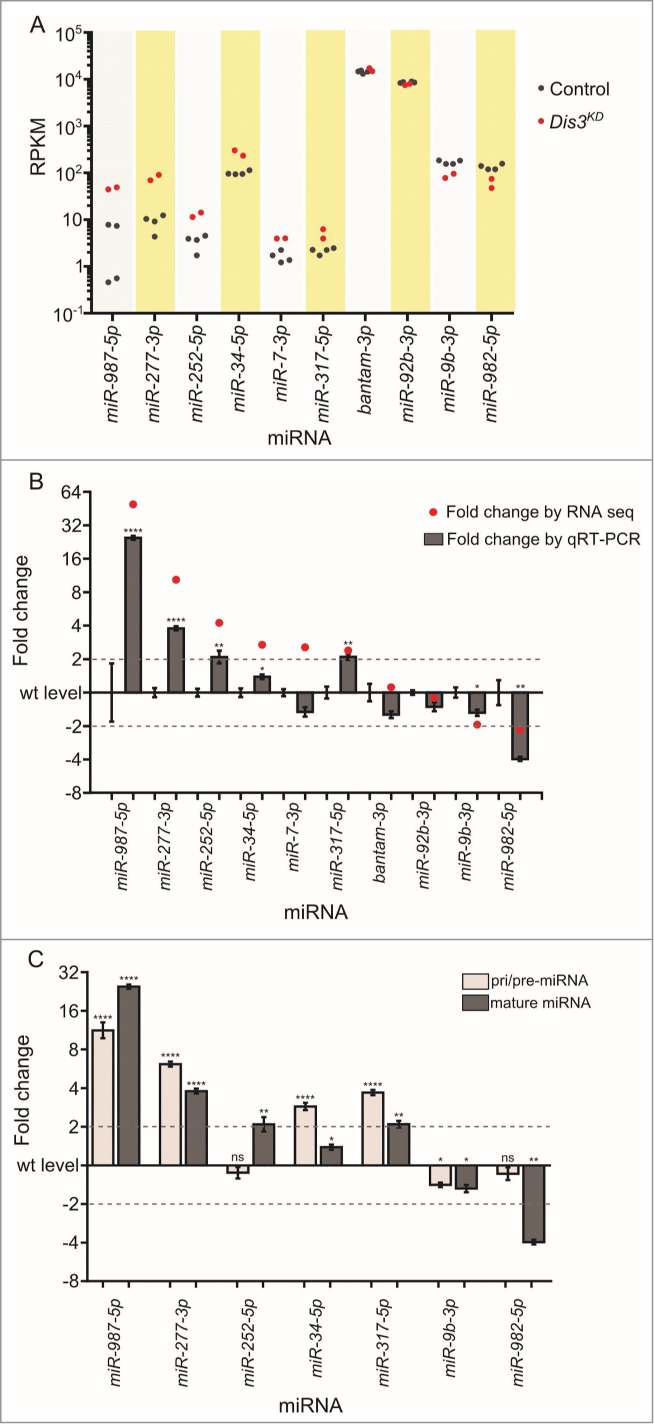
qRT-PCR validation of missexpressed miRNAs from the miRNA sequencing data. (**A**) Grouping of RPKM of selected miRNAs for all replicates is highly consistent between controls (black dots) and knockdown replicates (red dots). (**B**) Validation of miRNA-seq fold changes (red dots) using qRT-PCR. All selected miRNAs that change in expression upon Dis3-depletion in the miRNA-seq data also significantly change in expression when using qRT-PCR (except *miR-7–3p*). Dotted lines show +/−2-fold changes. Stars represent levels of significance calculated using a 2 sample t-test. (****p < 0.0001, **p < 0.01, *p < 0.05 n ≥ 3, error bars represent standard error). (**C**) The pri/pre-miRNA levels were measured for the miRNAs that significantly change in expression in both the miRNA-seq and qRT-PCR data. The expression levels of *miR-252–5p* and *miR-982–5p* change post-transcriptionally following Dis3 knockdown. All other miRNAs alter in a transcriptional manner as the pri/pre-mRNA (light gray) also changes significantly in expression, similar to the levels observed mature miRNAs (dark gray). Dotted lines show +/−2-fold changes. Stars represent levels of significance calculated using a 2 sample t-test. ****p < 0.0001, **p < 0.01, *p < 0.05, ns p > 0.05. (n ≥ 3, error bars represent standard error).

In order to validate the changes in miRNA abundance in wing imaginal discs following Dis3 depletion, we employed qRT-PCR to confirm the fold changes shown by RNA-seq. The first criterion for choosing this set of miRNAs was that they should include only those that showed changes in levels of ≥2-fold in both knockdown samples versus all control replicates. The second criterion was that the RNA-seq read counts should show consistency between the knockdown samples and between each of the parental control replicates (see Materials and Methods for further information). **Figure 6A** graphically represents the variation between replicates, as well as the abundance of miRNAs in terms of RPKM. Using this stringent filtering method, we identified 6 miRNAs whose expression increased ≥2-fold in the knockdown samples compared to both parental controls (*miR-277–3p, miR-987–5p, miR-252–5p, miR-34–5p*, and *miR-7–3p* and *miR-317–5p*). Two miRNAs whose expression was reduced ≥2-fold (*miR-982–5p* and *miR-9b-3p*) were also identified (**Figs. 4 and 6A**). Two miRNAs, *bantam-3p* and *miR-92b-3p* were chosen as controls as their levels remained the same in knockdown and parental samples.

The changes in expression of these miRNAs in Dis3-depleted wing imaginal discs compared to parental controls were validated using TaqMan qRT-PCR with *snoR442* as a normalization control. Expression of each miRNA was tested in at least 3 biological replicates with 4 technical replicates in total per biological replicate (see Materials and Methods). **Figure 6B** shows that the changes in expression levels for all selected miRNAs, as measured by qRT-PCR, were comparable with the levels determined by RNA-seq, apart from for *miR-7–3p* which had a very low normalized read count. The two control miRNAs, *bantam-3p* and *miR-92b-3p*, whose expression levels remained consistent across all samples according to RNA-seq data, also showed no significant changes as determined by qRT-PCR. Therefore, these data show that the changes in abundance of miRNAs upon Dis3 depletion, as measured by qRT-PCR, are similar to those determined by RNA-seq.

### Mature *miR-252–5p* is a potential target for specific Dis3 degradation

If miRNAs increase in expression in Dis3 knockdown cells then this could be due to the miRNA normally being targeted for degradation by Dis3. Alternatively, upregulation of the level of a miRNA could be a result of Dis3 knockdown indirectly upregulating the transcription of that miRNA. To test this we designed TaqMan assays specific to each of the pri/pre-miRNA precursors for each upregulated miRNA and compared the expression with that of mature miRNAs. Mature *miR-252–5p* appears to be a potential target of Dis3 because the levels of the *pri/pre-miR-252* precursor are similar in Dis3-depleted cells and controls whereas the mature *miR-252–5p* levels are 2.1-fold higher in depleted cells compared to controls (by qRT-PCR; **Fig. 6C**). In contrast, the levels of the pri/pre-miRNAs are increased in levels in Dis3-depleted cells for *miR-277–3p, miR-34–5p, miR-317–5p and miR-987–5p* suggesting that these miRNAs are not direct targets of Dis3.

Levels of *pre-miR-982–5p* are similar in Dis3-depleted cells compared to controls whereas the mature *miR-982–5p* decreases 4.4-fold (by qRT-PCR). This suggests that Dis3 is involved in the processing of the mature *miR-982–5p* from the *pre-miR-982* precursor. For *miR-9b-3p*, the levels of *pre-miR-9b* show a similar decrease to that of the mature miRNA suggesting that this miRNA is indirectly regulated by Dis3, perhaps by indirectly affecting transcription.

### Overexpression of *miR-252–5p* in the wing pouch results in severe wing defects

**Figure 7.**
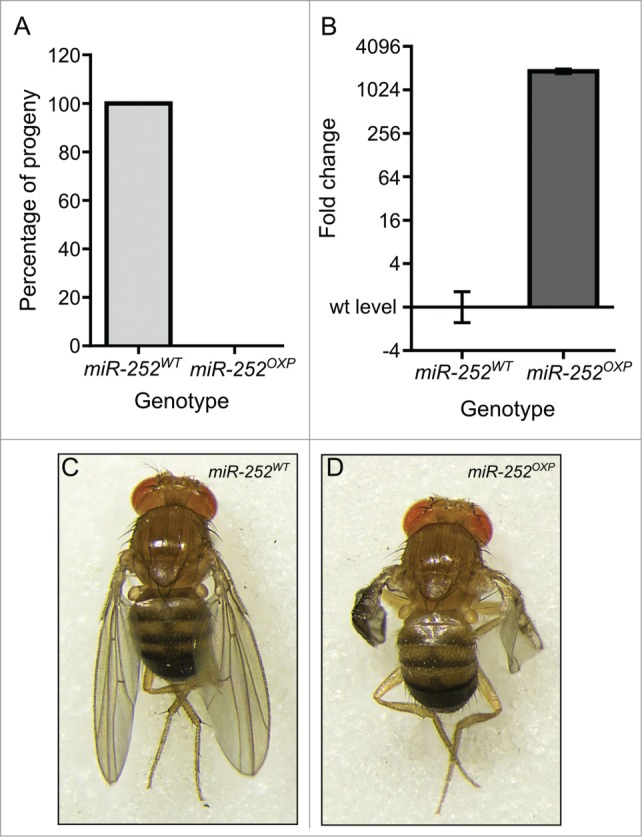
Overexpression of *miR-252* in the wing pouch of the imaginal disc results in severe wing phenotypes. (**A**) Overexpression of *miR-252* using the ubiquitously expressed *tub-GAL4* driver results in 100% lethality compared to controls (*UAS-miR-252/TM6*). n = 184. (**B**) Overexpression of *UAS-miR-252* in the wing pouch using the *nubbin-GAL4* driver results in a 1154-fold increase in *miR-252–5p* expression in the wing imaginal disc. (n ≥ 3, *p* < 0.0001, error bars represent standard error). (**C and D**) Overexpression of *UAS-miR-252* in the wing pouch of the wing imaginal disc using the *nubbin-GAL4* driver (*;nub-Gal4/+ ;UAS-miR-252/+*) results in severe wing crumpling (**D**) compared to a parental control (*miR-252*^*WT*^ is *UAS-miR-252*) (**C**) with 100% penetrance. n = 168.

If Dis3 normally affects wing growth by repressing the expression of *miR-252–5p*, then we would expect that overexpression should result in similar phenotypes to knockdown of Dis3. To test this, we first overexpressed *UAS-miR-252* pre-miRNA under the control of the *tubulin-GAL4* promoter. Similarly to the Dis3 knockdown, this resulted in complete lethality (**Fig. 7A**). We then overexpressed *UAS-miR-252* in the wing pouch using the *nubbin-GAL4* driver. As expected, mature *miR-252–5p* was highly expressed (1154-fold increase) in the wing imaginal discs of *nub-GAL4/UAS-miR-252* larvae (**Fig. 7B**). Overexpression of *miR-252* resulted in a similar but less severe phenotype to that seen in surviving Dis3 knockdown flies (100% penetrance) (compare **Fig. 7C,D** with **Fig. 1A,B**). Therefore it is possible that Dis3 normally targets *miR-252–5p* to keep its expression at low levels and prevent it from inhibiting wing development.

### Knockdown of Rrp40, an exosome subunit, results in upregulation of *miR-252–5p*

**Figure 8.**
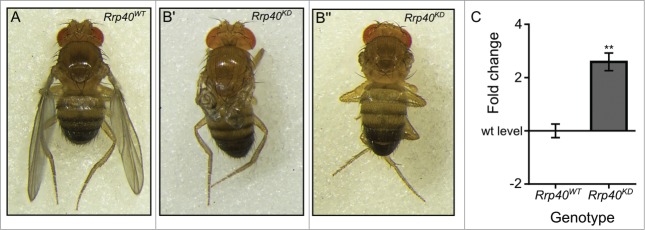
Knockdown of the exosome subunit Rrp40 results in similar phenotypes as to Dis3 knockdown. Knockdown of the exosome subunit Rrp40 using the *nubbin-GAL4* wing pouch driver (*;nub-Gal4/UAS-Rrp40*^*RNAi*^;) results in severe wing development phenotypes (B' and B”) when compared to parental controls (**A**). 90% of adults present a severe phenotype with crumpled wings (**B'**) and the remaining 10% show a similar phenotype to Dis3 knockdown with no wings (B”). (**C**) Knockdown of Rrp40 in the wing pouch (*nub-GAL4*) results in a similar increase in *miR-252–5p* expression as observed during Dis3 depletion, indicating the effect observed upon Dis3 knockdown is a result of Dis3/exosome impairment (n≥3, **p < 0.01, error bars represent standard error).

In *Drosophila*, the exosome subunit Rrp40 has been shown to be predominantly localized to the cytoplasm.^35^ To determine if this exosome subunit is involved in the degradation of *miR-252–5p*, or whether Dis3 acts independently of this exosome subunit, we knocked down Rrp40 in the wing pouch using the *nubbin-GAL4* driver. As can be seen in **Figure 8A-B** this knockdown resulted in phenotypes similar to, but less severe than, phenotypes seen for Dis3 knockdown. In addition, the levels of mature *miR-252–5p* increased 2.5-fold, which is comparable to the 2.1-fold increase seen in Dis3-depleted cells (**Fig. 8C**). This result is consistent with Dis3 acting in a complex with Rrp40 in the control of the levels of *miR-252–5p* in the cytoplasm.

## Discussion

In this study, we have used RNA-seq to investigate the effects of knockdown of the 3′-5′ exoribonuclease Dis3 on the levels of miRNAs in *Drosophila* wing imaginal discs. We show that the expression of a small subset (17/109) are changed ≥2-fold upon Dis3 depletion. For two of the miRNAs, the changes in abundances of the mature miRNAs are not accompanied by changes in the levels of the precursor miRNAs, suggesting regulation at the post-transcriptional level. For the upregulated mature miRNA, *miR-252–5p*, this suggests that Dis3 can selectively degrade this miRNA in the cytoplasm. For the downregulated miRNA, *miR-982–5p*, our data are consistent with a role of Dis3 in the processing of the miRNA from the pre-miRNA precursor. We also show that overexpression of *miR-252–5p* produces a similar phenotype to knockdown of Dis3, demonstrating that regulation of this miRNA is important in wing development. Knockdown of the predominantly cytoplasmic exosome subunit Rrp40 also results in an upregulation of *miR-252–5p* and a similar but less severe phenotype than Dis3 depletion, suggesting that the exosome is at least partially required for the activity of Dis3 over this miRNA. This study therefore reveals, for the first time, that Dis3 appears to have important biological roles in the processing of specific mature miRNAs in the cytoplasm.

The use of RNA-seq as an unbiased approach to detect miRNAs in wing imaginal discs has also allowed us to assess the abundance of individual miRNAs within wing imaginal discs (rather than all imaginal discs) from larvae at the L3 stage. Our data show that the most abundant miRNA, *miR-9a-5p*, is about 12,000-fold more abundant than the levels of the least abundant miRNA reliably detected (*miR-307a-3p*). The abundance profile of miRNAs is in line with the profile determined by microarrays,^5^ although more miRNAs are detected by the more sensitive RNA-seq technique, as expected. Analysis of the RNA-seq data also identified a novel *Drosophila* miRNA, which is conserved in the red flour beetle, *Tribolium castaneum*. This analysis also shows that the miRNAs which are upregulated upon Dis3 depletion tend to be poorly expressed in wild-type discs, suggesting that Dis3 is normally required to maintain these miRNAs at low levels, at least during this stage of development.

The set of miRNAs that increase ≥2-fold; by RNA-seq upon Dis3 depletion are *miR-277–3p, miR-987–5p, miR-252–5p, miR-34–5p* and *miR-317–5p* (**Table 2**). For *miR-277–3p, miR-987–5p* and *miR-34–5p* their cognate miRNA precursors also increase upon Dis3 knockdown, suggesting that Dis3 normally affects their expression via indirect means, perhaps by increasing the expression of specific transcription factors. Details on the known biological functions of these miRNAs in *Drosophila* are given in **Table 2**. In contrast, the expression of *pre-miR-252* does not increase upon Dis3 knockdown whereas the mature miRNA increases by 2.1-fold (by qRT-PCR). This suggests that Dis3 normally degrades this miRNA in the cytoplasm. *miR-252* is conserved in all *Drosophila* species and also in invertebrates (**Table 2**) suggesting that it has important biological functions. Overexpression of *miR-252* in the wing pouch gives a similar, although weaker phenotype to Dis3 knockdown, which is expected if it is a target of Dis3. This data is consistent with previous results showing that ectopic expression of *miR-252* in the wing pouch using the *scalloped*-*Gal4* driver also induced strong wing phenotypes.^36^ In addition, it has been reported that a severe wing phenotype is observed when *miR-252–5p* is overexpressed using the *MS1096-GAL4* driver.^37^ Wing phenotypes are not always seen when miRNAs are overexpressed in the wing; for example, no phenotype is seen when *miR-34* is overexpressed using *MS1096-GAL4*.^37^ No targets of *miR-252–5p* have been determined experimentally, but predicted targets include mRNAs important in developmental processes such as *ultrabithorax* and *smoothened* (http://www.targetscan.org/fly_12/).

**Table 2.** Summary of missexpressed miRNAs in Dis3 knockdown wing imaginal discs

miRNA	Fold changeSeq/qRT-PCR	Conservation	Known function in *Drosophila*	Validated targets in *Drosophila*	Clustering (same transcriptional unit)	References
*miR-987–5p*	+49.5/+16.8	Dm	Unknown	None identified	No	
*miR-277–3p*	+10.4/+3.7	Dm	Controls valine leucine and isoleucine degradation	None identified	*miR-34*	^48,49^
*miR-252–5p*	+4.2/+2.1	Sp, Dm, Ce,	Misregulated in stress and dystrophy	None identified	No	^50^
*miR-34–5p*	+2.7/+1.4	Hs, Sp, Dm, Ce	Negative regulation of neuronal cell death	*Su(z)12, Eip74EF*	*miR-277*	^51,52^
*miR-317–5p*	+2.4/2.1	D	Brain morphogenesis	None identified	No	^53^
*miR-9b-3p*	−2.0/−1.5	Hs, Sp, Dm, Ce,	Unknown	None identified	*miR-9c, miR-306* and *miR-79*.	
*miR-982–5p*	−2.2/−4.4	Dm	Unknown	None identified	No	

Hs – *Homo sapiens*, Sp – *Strongylocentrotus purpuratus*, Dm – *Drosophila melanogaster*, Ce – *Caenorhabditis elegans*. *miR-317 is located in close proximity to *miR-34* and *miR-277*. **miR-982 is located in close proximity to *miR-984, miR-303, miR-983–1* and *miR-983–2*. Conservation taken from miRBase release 19.

Of all the miRNAs detected by RNA-seq in Dis3 depleted wing imaginal discs, only one, *miR-982–5p*, showed a decrease in level (-4.4-fold by qRT-PCR) while its precursor remained unchanged. These data suggest that Dis3 is involved in the processing of the mature miRNA from its precursor miRNA. Although the qRT-PCR assays cannot distinguish the pri-miRNA precursor from the pre-miRNA precursor, our RNA-seq data shows that the *miR-982–3p* reads decrease to a similar extent to the *miR-982–5p* reads. This data supports the suggestion that Dis3 is involved in processing the pre-miRNA precursor from the pri-miRNA precursor. The biological function of *miR-982–5p* is not known but ectopic expression of *pre-miR-982* in the wing pouch using the *scalloped-GAL4* driver has been shown to produce phenotypes consistent with a connection to Notch signaling.^36^

The above mechanism of regulation relies on a percentage of Dis3 protein being located in the cytoplasm. Our previous published work on ovaries and embryos, and that of others using S2 cells, has shown that Dis3 is predominantly located in the nucleus^7,19,20^ with about 10% being located in the cytoplasm.^19^ Therefore, there would appear to be sufficient Dis3 in the cytoplasm to carry out the activities observed. Since the upregulation of *miR-252–5p* in Dis3-depleted cells is similar to that in Rrp40-depleted discs it is likely that it is acting together with the core exosome complex rather than acting independently or in sub-complexes.^32^ Rrp40 has been shown to be predominantly localized to the cytoplasm in *Drosophila*,^35^ therefore this would be consistent with the cytoplasmic exosome being responsible for the degradation of *miR-252–5p*. The phenotypes resulting from Rrp40 knockdown are also similar to Dis3 knockdown which is also consistent with Dis3 acting together with the core exosome complex.

An interesting feature of our research is the co-ordinate regulation of 2 miRNAs by the 3′-5′ and 5′-3′ degradation pathways. *miR-277–3p* increases 3.8-fold (by qRT-PCR, **Fig. 6C**) upon Dis3 knockdown, with this increase most likely to be due to an indirect effect on transcription. In contrast, in the hypomorphic *pcm*^5^ mutant, where activity of the 5′-3′ exoribonuclease Pacman (Xrn1) is decreased, mature *miR-277–3p* is decreased in expression by 5.6-fold, while the level of *pre-miR-277–3p* remains unchanged.^5^ These data therefore suggest that both Pacman and Dis3 are required to maintain this miRNA at the correct level in the wing imaginal discs. Therefore the normal expression of *miR-277–3p* would be achieved through a balance between upregulation by Pacman (perhaps due to increased processing from precursors) and downregulation by Dis3 (most likely due to indirect transcriptional effects). A similar effect is seen for *miR-34–5p* although the changes in levels are not so pronounced. The exact mechanistic pathways whereby this is achieved remains to be determined.

What are the mechanisms by which *miR-252–5p* may be targeted to Dis3 for degradation? One possibility is that this miRNA is selectively uridylated or adenylated at the 3′ end to promote degradation by Dis3. Adenylation by Wispy has been clearly shown to result in decreased stability of *miR-312* in *Drosophila* cells,^38^ however, the exoribonuclease responsible for the degradation has not been determined. It is therefore possible that *miR-252–5p* is normally adenylated (or possibly uridylated) at the 3′ end by a nucleotidyl transferase to promote its degradation. It has also been reported that *Drosophila* miRNAs may be modified by 3′ trimming by the exonuclease Nibbler.^39^ As well as enhancing/inhibiting exonuclease activity these 3′ end modifications may alter affinity for binding proteins such as AGO in the RISC and so promote or prevent release of the miRNA from the protein complex. However, our RNA-seq data shows no clear evidence for uridylation, adenylation or 3′ trimming of this miRNA. One reason for this is that the 2 bases 3′ to the canonical mature *miR-252–5p* sequence, as encoded by the DNA sequence, are uridines. Therefore we cannot distinguish whether the first and/or second uridines at the 3′ end of some of the reads are a result of post-transcriptional additions (i.e. non-templated) or are encoded by the DNA template (i.e., templated). Similarly, *miR-982–5p* also has a uridine encoded at the next nucleotide after the 3′ end of the mature miRNA in the DNA sequence. In addition, size selection of RNAs for the RNA-seq ranged from 18–22nt therefore there is very little scope for detection of non-templated nucleotides at the 3′ ends of the RNA reads. Another way to promote miRNA instability could be the binding of *miR-252–5p* to its mRNA targets or to long non-coding targets.^40,41^

Our data also suggest that Dis3 can specifically affect processing of *miR-982–5p* from its precursors. This specificity could be conferred by preferential binding of Dis3 to pre-miRNAs with >2nt 3′ overhangs plus binding and co-operation with the terminal uridyl transferases TUT7 and TUT4 as occurs in human cells.^29^ An alternative scenario is that *pre-miR-982* binds to particular RNA binding proteins (e.g. Loquacious (PACT in mammals)^42,43^) to enable trimming of the precursor by Dis3. If Dis3 is involved in the processing of *pre-miR-982* during its biogenesis then in the absence of Dis3 one may expect the level of precursor to increase. However, our data shows that *pre-miR-982* remains unchanged following Dis3 knockdown. The reasons for this may be twofold; firstly in the absence of Dis3, *pre-miR-982* may be signaled for degradation by another 3′-5′ exoribonuclease, for example, by addition of uridyl residues to the 3′ end and degradation by Dis3L2 in a similar manner to that observed during *let-7* biogenesis in mouse and human cells.^16,18^ Alternatively Dis3 could function in the final trimming of *miR-982–5p* similar to the activity observed for Nibbler.^39,44^ In this case the *pre-miR-982* would be processed as normal by Dicer and therefore no change in *pre-miR-982* levels would be expected. Our findings set the stage for further mechanistic studies to understand the specificity of Dis3 in the control of miRNAs biogenesis and degradation.

## Materials and Methods

### Fly stocks, crosses and phenotypic analyses

Fly stocks were cultivated on standard media at 25°C in uncrowded conditions. All stocks used were obtained from the Bloomington stock center unless otherwise stated. GAL4 drivers used were: *tub-GAL4* (*P{w*^*+mC*^*=tubP -GAL4}LL7* originally from stock 5138, *y*^*1*^ w* *;; P{w*^*+mC*^*=tubP -GAL4}LL7/TM6b,GFP*), *da-GAL4* (stock 108252, *w*^*1118*^*, P{w*^*mW.hs*^*Gal4-da.G32}UH1*), *nub-GAL4* (stock 25754; *P{UAS-Dcr-2.D}1, w*^*1118*^*; P{GawB}nub-AC-62*), *69B-GAL4* (stock 1774; w*;; P{GawB}69B) and *pnr-GAL4* (stock 25758, *P{UAS-Dcr-2.D}1, w*^*1118*^*; P{GawB}pnr*^*MD237*^*/TM3*). *UAS-miR-252* was obtained from FlyORF (stock F002053, *M{UAS-miR-252.S}ZH-86Fb*). RNAi stocks were obtained from the Vienna Drosophila RNAi Center. Stocks used were: *UAS-Dis3*^*RNAi*^ (stock v35090, *w*^*1118*^ *;; P{GD11917}v35090* and stock v108013, *; P{KK101473}VIE-260B;, UAS-Rrp40*^*RNAi*^ (stock v40306, *w^1118^; P{GD10414}v40306;*). Phenotypes produced by both *UAS-Dis3^RNAi^* lines are indistinguishable from each other when driven by all drivers except for *69B-GAL4*, where lethality occurs at a slightly later stage (L3) for v108013. Images of adult flies were obtained by freezing the flies at −20°C for 15 minutes and then capturing a series of images at different focal depths using a Nikon D5200 camera mounted on a Nikon SMZ800 dissecting microscope. The series of images were stacked and merged in Adobe Photoshop CS6 producing the final image. For the measurement of developmental delay, crosses were set up at 25°C with flies left for 8 hours to lay eggs (8hr egg lay) with time 0 starting after the 8 hours. Times were recorded when progeny reached wandering L3, pupal and adult stages of development.

### Measurement wing imaginal disc sizes

Wing imaginal discs were dissected from late L3 larvae in PBS. Late L3 larvae were staged by the addition of 0.05% bromophenol blue to the food and selecting only larvae that had cleared the dyed food from their gut. Dissected discs were mounted on poly-l-lysine slides in 85% glycerol. Disc area was measured using Axiovision 4.7 on an Axioplan microscope (Carl Zeiss).

### Western blotting

Western blotting was performed on samples containing 60 wing imaginal discs. Tubulin was used as a loading control. Mouse anti-Tubulin primary antibody (Sigma, cat. no. T9026) was used at a 1:2,000 dilution with an anti-mouse-HRP conjugated secondary antibody (Sigma, cat. no. A2304) at 1:80,000. Rabbit anti-Dis3^7^ was used at 1:1,500 with an anti-rabbit-HRP conjugated secondary antibody (Sigma, cat. no. 1949) at 1:80,000. Antibody binding was detected using Amersham ECL detection reagents (GE Healthcare, cat. no. RPN2209). Relative quantification of bands was performed with ImageJ software.

### Immunocytochemistry

Immunocytochemistry was performed essentially as described in.^45^ Images were taken with a Zeiss Axiovert confocal microscope equipped with a LSM520 Meta. Anti-Cleaved Caspase-3 (Asp175) (Cell Signaling, cat no. 9661) was used at 1:400 dilution. Cy3-conjugated monoclonal donkey anti-mouse IgG secondary antibody was used at 1:400 (Jackson ImmunoResearch, cat. no.715–165–151).

### RNA extraction and qRT-PCR

RNA extractions were performed using a miRNeasy RNA extraction kit (Qiagen, cat. no. 217084), with an on-column DNase digestion (Qiagen, cat. no. 79254). RNA concentrations were measured on a NanoDrop 1000 spectrophotometer (Thermo Scientific). For qRT-PCR, RNA samples were diluted to a consistent concentration then cDNA was prepared in duplicate using a High Capacity cDNA Reverse Transcription Kit (Life Technologies, cat. no. 4368814) with random primers. A control “no RT” reaction was performed in parallel to confirm that all genomic DNA had been degraded. qRT-PCR was performed on each cDNA replicate in duplicate (i.e. 4 technical replicates in total), using TaqMan Universal PCR Master Mix, No AmpErase UNG (Life Technologies, cat. no. 4324018) and an appropriate custom designed TaqMan pre-miRNA assay (Life Technologies). For custom pre-miRNA assays, the pre-miRNA hairpin sequence (miRBase) was submitted to Life Technologies' web-based Custom TaqMan Assay Design Tool as in^5^ (**Fig. S4**). *RpL32* (*Rp49*) was used for normalization. For miRNA qRT-PCR, RNA was diluted to 2 ng/μl then cDNA was prepared in duplicate from 10ng RNA using a TaqMan miRNA Reverse Transcription Kit (Life Technologies, cat. no. 4366596) with miRNA specific primers. As above a no RT reaction was performed in parallel. qRT-PCR was performed as above. *snoR442* was used for normalization.

### Small RNA sequencing

RNA extraction was performed as above from 60 wandering L3 wing imaginal discs. RNA integrity was checked using an Agilent 2100 Bioanalyser. 1μg total RNA was sent to ARK genomics for sample preparation and sequencing. Libraries were prepared using an Illumina TruSeq small RNA kit and size selected to specifically contain RNAs of 18–22 bp in length. Samples were run on a HiSeq 2500 to give between 12 and 22 million reads per sample. Analysis was completed by ARK Genomics. Adaptors were removed using Cutadapt.^46^ The remaining reads were mapped to miRBase release19 using Bowtie 2.^47^ Mapped reads were normalized per million reads (RPKM). The total number of reads per sample ranged between 12 and 22 million, of which on average 1.12% mapped to mature miRNAs. This low percentage of miRNA mapped reads was due to the presence of 2S rRNA which would have been retained during the size selection. miRNAs that varied significantly between parental lines were excluded from further analysis (outliers in **Fig. 3C**). In addition we also removed any miRNAs whose expression differed more than 2-fold between biological replicates of the same genotype. Finally we only selected miRNAs that changed in expression under the test conditions more than 2-fold against all parental replicates for further analysis. One miRNA was selected that did not pass these filters. *miR-987–5p* expression varied between parental controls, however due to it showing the greatest level of upregulation following Dis3 knockdown against both separate parents, it was taken for further analysis. Due to the overall similarity between the 2 parental controls (**Fig. 3C**) we were able to group them together into an overall 'control' group. All comparisons shown are between the Dis3 knockdown samples and the grouped parental controls.

### Identification of known and novel miRNAs

Fastq files were converted to fasta files using the FASTX toolkit (http://hannonlab.cshl.edu/fastx_toolkit/). Reads were simultaneously clipped and mapped using the mapper.pl script included in miRDeep2.^34^ Reads were mapped to the *Drosophila melanogaster* genome, version BDGP5 (Ensembl) (Settings:-c –j –k –I 17 –m). To identify novel miRNAs miRDeep2.pl was used. *Drosophila simulans, Drosophila sechellia, Drosophila erecta, Drosophila yakuba and Drosophila ananassae* were used as similar species to aid the identification of novel miRNAs. Potential novel miRNAs were selected for further analysis if they were found in at least 5 of the 6 replicates, including at least one of each genotype. The novel miRNA *miR-11182-3p* was validated using a custom TaqMan assays to the identified sequence (CACAUCGAUGUUUUUCCACCUC). qRT-PCR was completed with the custom assay as above. The secondary structure of the novel miRNA was predicted using RNAfold WebServer (http://rna.tbi.univie.ac.at/cgi-bin/RNAfold.cgi) with default settings.

### Statistical analyses

All statistical analyses were performed in GraphPad Prism 6 except for density and scatter plots which were produced in R (version 3.1.1). All data analyzed were compatible with parametric tests. Two-sided 2-sample t-tests were used to compare the means of single test groups to single control groups. If multiple comparisons were required, a one-way ANOVA was performed with a post-test to compare the means of each possible pair of samples.

## Note

Raw sequencing data can be accessed from Array Express under the accession number E-MTAB-3569 (http://www.ebi.ac.uk/arrayexpress/experiments/E-MTAB-3569/).
