# Supplementary material for: The 3'-5' exoribonuclease Dis3 regulates the expression of specific microRNAs in Drosophila wing imaginal discs
Source: RNA Biol. 2015 Apr 18;12(7):728–41. doi: 10.1080/15476286.2015.1040978 (PMC4615222; doi:10.1080/15476286.2015.1040978)
Supplement: Supplemental_Figures.zip [file krnb-12-07-1040978-s001.zip › Supplemental Figure 4.docx]

*pre-miR-277* Forward: 5’-AGGTGAGTGTCTAGTGAAGCATCTA-3’

Probe: 5’-TAGTGTCTCCGTAAACTTTT-3’

Reverse: 5’-ACGAGAGAAACAGCTAGA-3’

*pre-miR-987* Forward: 5’-TGTTGGACTGTGTTTAAAGTAAATAGTCTG-3’

Probe: 5’-CTCGAATGCAACTTTC-3’

Reverse: 5’-GCAGTTGAAGTAAATGCCTGTTGAT-3’

*pre-miR-252* Forward: 5’-ACCAAGTTCGCTTTCCTAAGTACTAG-3’

Probe: 5’-TTGCGGACACGAACCTA-3’

Reverse: 5’-CTTGGGCAGCAGGAGGTA-3’

*pre-miR-34* Forward: 5’-AGTGTGGTTAGCTGGTTGTGTAG-3’

Probe: 5’-CAACGGCAATAATTG-3’

Reverse: 5’-AGTGAAGATAGTGGCTGTGAATTGT-3’

*pre-miR-317* Forward: 5’-ACCCTGTGCTCGCTTTGAA-3’

Probe: 5’-CACTTGCTTGCATTTCA-3’

Reverse: 5’-CAAACGGCCACTGGATACCA-3’

*pre-miR-982* Forward: 5’-CGAAATCATGTTAGATCCTGGACAAAT-3’

Probe: 5’-AAGTAATTGATGCATAAAAAC-3’

Reverse: 5’-GCTCTAAAACCATTTAGTTCAAGGATGAA-3’

*pre-miR-9b* Forward: 5’-TGCATATTATTTGCTCTTTGGTGATTTTAG-3’

Probe: 5’-CTGTATGGTGTTTATGTATATTC-3’

Reverse: 5’-TGCAGAAACCATTTGGTTTTTGGTA-3’
