# Supplementary figures and images for: The 3'-5' exoribonuclease Dis3 regulates the expression of specific microRNAs in Drosophila wing imaginal discs
Source: RNA Biol. 2015 Apr 18;12(7):728–41. doi: 10.1080/15476286.2015.1040978 (PMC4615222; doi:10.1080/15476286.2015.1040978)

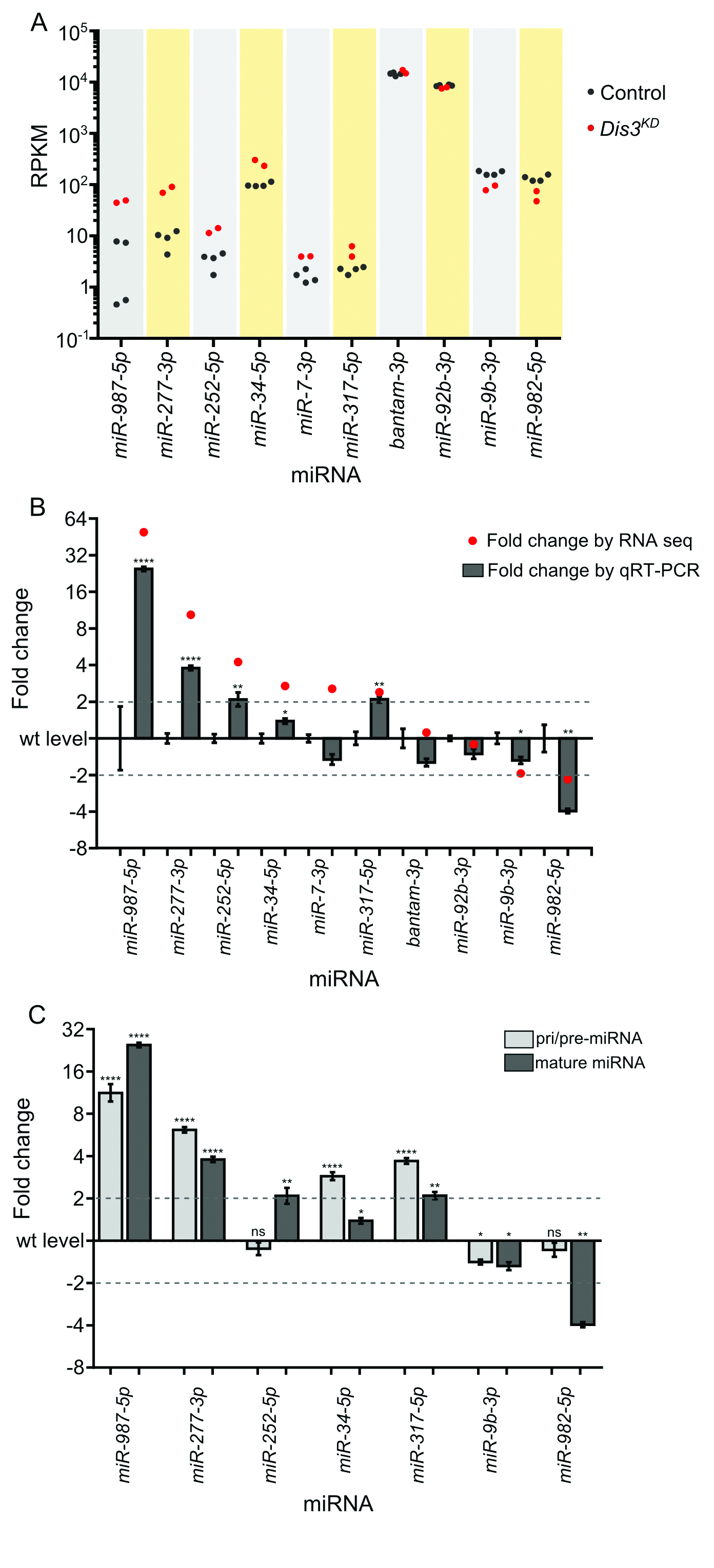

Supplement: Supplemental_Figures.zip [file krnb-12-07-1040978-s001.zip › Supplemental Figure 1.jpg]

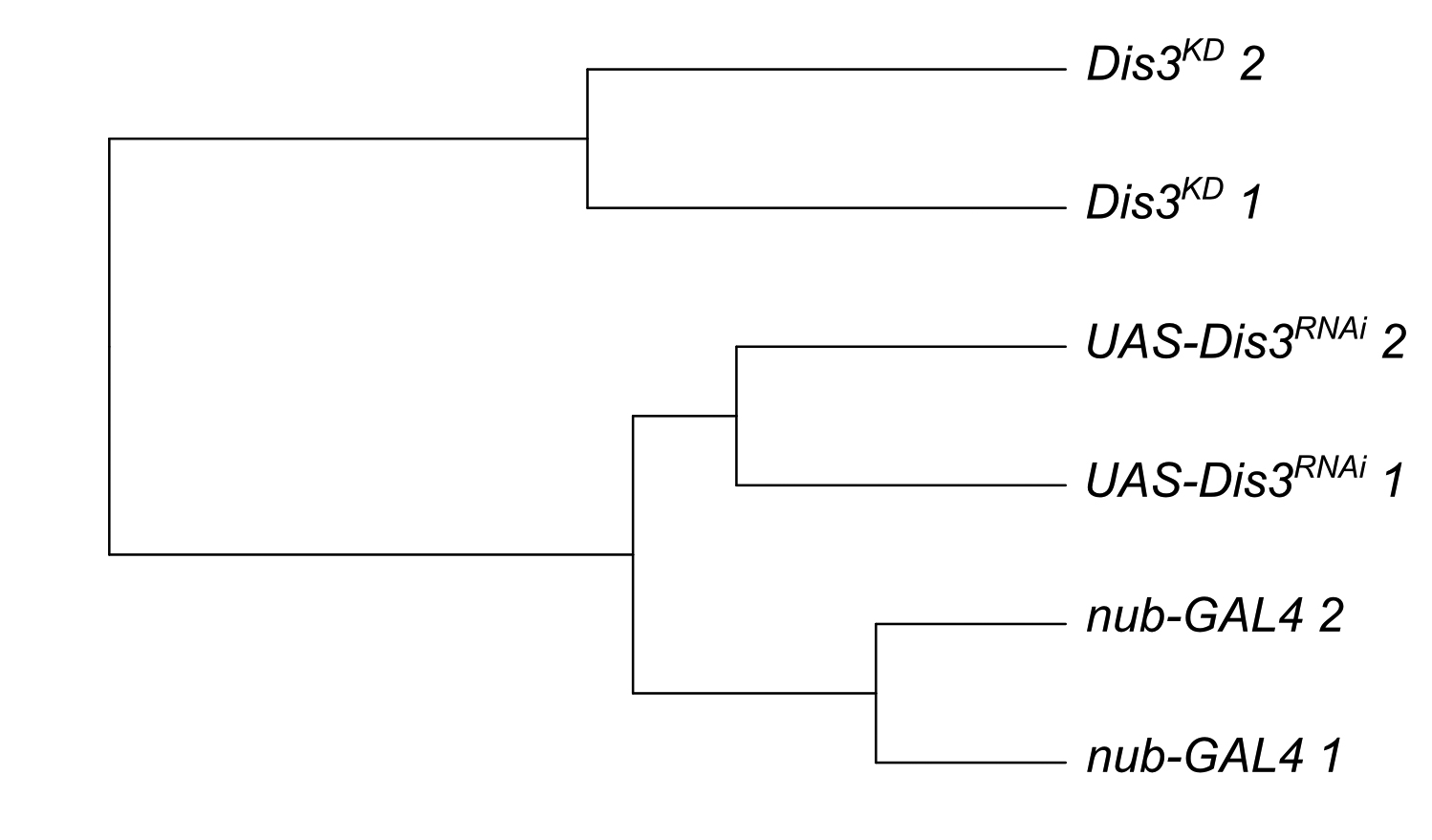

Supplement: Supplemental_Figures.zip [file krnb-12-07-1040978-s001.zip › Supplemental Figure 2.jpg]

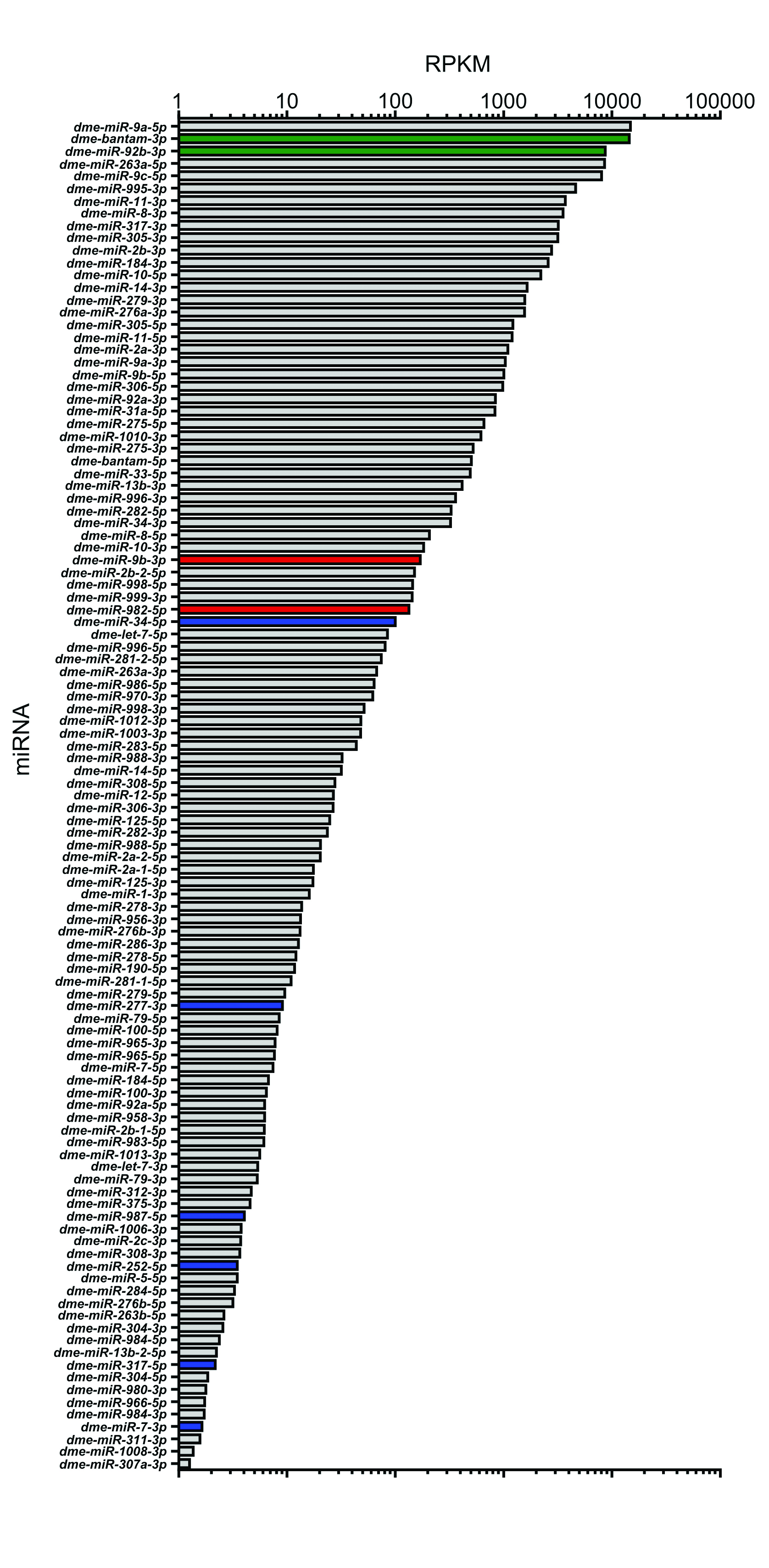

Supplement: Supplemental_Figures.zip [file krnb-12-07-1040978-s001.zip › Supplemental Figure 3.jpg]
